# Comparison of Frailty Criteria, Cognitive Function, Depressive and Insomnia Symptoms in Men with Localized and Advanced Prostate Cancer under Androgen Deprivation Therapy

**DOI:** 10.3390/healthcare11091266

**Published:** 2023-04-28

**Authors:** Mayra Alejandra Mafla-España, María Dolores Torregrosa, Manel Beamud-Cortés, Lorena Bermell-Marco, José Rubio-Briones, Omar Cauli

**Affiliations:** 1 Nursing Department, University of Valencia, 46010 Valencia, Spain; 2 Frailty Research Organized Group (FROG), University of Valencia, 46010 Valencia, Spain; 3 Medical Oncology Department, Doctor Peset University Hospital, 46017 Valencia, Spain; 4 Urology Department, Doctor Peset University Hospital, 46017 Valencia, Spain; 5 Urology Clinic, Hospital VITHAS 9 de Octubre, 46015 Valencia, Spain; 6 Chair of Healthy, Active and Participative Ageing, University of Valencia, 46010 Valencia, Spain

**Keywords:** frailty syndrome, geriatric assessment, LHRH analogues, symptoms of insomnia, metastatic prostate cancer, localized prostate cancer

## Abstract

Background: Prostate cancer (PCa) is considered one of the most important medical problems in the male population, with a very high incidence after the age of 65. Frailty represents one of the most critical issues facing healthcare due to its inherent relationship with poor healthcare outcomes. The physical phenotype of frailty syndrome based on Fried criteria has been associated with poor outcomes, morbidity, and premature mortality. To date, there are few studies that have analyzed frailty syndrome in patients with localized and advanced (mPCa) disease under androgen-deprivation therapy. Objective: Our goal was to assess whether there are differences in frailty criteria between mPCa and localized PCa. We also evaluated the role of other geriatric variables such as depressive and insomnia symptoms, which are frequently reported in cancer patients. Methods: In this cross-sectional study, frailty syndrome was evaluated in both groups, as well as its possible relationship with cognitive functions, depressive and insomnia symptoms, and other clinical variables related to PCa and its treatment. Frailty was defined on Fried’s criteria: low lean mass, weakness, self-reported exhaustion, low activity level, and slow walking speed; prefrailty was defined as having one or two of those criteria and frailty as having three or more, depressive symptoms were defined by the Yesavage scale, cognitive functions with the Mini-Mental examination test, and insomnia symptoms by the Athens scale and self-reported health status. Results: The prevalence of prefrailty/frailty was slightly higher in mPCa compared to localized PCa (81.5% versus 72.3%, respectively), however by analyzing each of the frailty criteria, two of them were significantly reduced in mPCa compared to localized PCa patients, e.g., gait speed (*p* = 0.001) and muscle strength (*p* = 0.04). The reduced gait speed and muscle strength in mPCa were not due to the increased age in mPCa group, or to an increase in comorbidities or shorter time under androgen-deprivation therapy. The symptoms of insomnia were significantly higher in mPCa patients compared to those with localized PCa (*p* < 0.05) whereas cognitive functions or depressive symptoms were not significantly different between the two groups. Conclusion: Patients with mPCa under androgen-deprivation therapy display higher alterations in gait speed and muscular strength and insomnia symptoms, thus interventions should be aimed to reduce these alterations in order to limit adverse outcomes related to them and to improve quality of life in these patients.

## 1. Introduction

Prostate cancer is considered one of the most important medical problems in the male population, with a very high incidence after the age of 65 [1]. With an estimated 1.4 million new cases and 375,000 deaths worldwide, prostate cancer was the second most common cancer and the fifth leading cause of cancer deaths among men in 2020 [2]. According to data reported by the Spanish Society of Medical Oncology, prostate cancer is the most diagnosed cancer in men in Spain, with 30,884 cases in 2022, and is the third most common cause of death [3]. While most cases follow an indolent course with no threat of mortality, many patients present with intermediate or high-risk localized, locally advanced, or metastatic cancer [4]. It has long been known that prostate cancer is unique in its dependence on androgens for growth and progression, and androgen deprivation is an effective therapeutic strategy that is widely used in clinical practice, allowing reduced tumor burden, improved survival, and palliation of symptoms [5]. Most patients with common malignancies are older adults. The physiological changes that occur during the aging process have a significant impact not only on the development and behavior of individual malignancies, but also on the physiological reserve and vulnerability of older patients who suffer from them, as well as from frailty syndrome [6], which is a state of extreme vulnerability resulting from the progressive decline in physiological systems associated with aging that leads to diminished reserves, so that the ability to cope with acute stressors is reduced and compromised [7]. Frailty is one of the most critical issues facing healthcare because of its inherent relationship with poor healthcare outcomes [8]. Given that both the cancer itself and the therapies offered can be significant additional stressors that challenge the patient’s physiological reserve, the incidence of frailty among older cancer patients is particularly high [9]. The onset of frailty syndrome with a lack of adherence to treatment [10,11], increased risk of falling [12,13], disability [14], dependency, need for long-term care [15], and death [16,17] may contribute to the development or progression of chronic diseases [18,19] and limit therapeutic options.

The geriatric assessment is a multidisciplinary diagnostic process of frail older people that assesses medical, psychological, social, and functional capacity [20]. The International Society for Geriatric Oncology (SIOG) established recommendations for geriatric evaluations in older cancer patients in 2005. Geriatric assessment is valuable in oncology due to its ability to detect various geriatric problems not recognized in clinical oncology care, its ability to predict the risk of treatment-related complications (e.g., chemotherapy toxicity and surgical risks) [21], and it can influence the choice of treatment and intensity [22]. Geriatric assessment necessarily includes the evaluation of the patient’s functional capacity and can be applied at any level of health, even at home; this allows a focus on biological, psychological and social aspects, as well as making possible the systematic observation of the therapeutic interventions, with the aim of preventing or at least delaying the onset of disabilities. The geriatric assessment includes at least an evaluation of functional status, cognition, and mood [23]. Functional status can be assessed with the frailty phenotype based on five physical criteria proposed by Fried et al., and is useful for identifying frail individuals [7]. Hormones are attractive candidates as contributors to the physiological dysregulation underlying frailty, because of their potential effects on multiple organ systems [24]. Several components of frailty syndrome are related to the physiological actions of androgens [25], and their deficiency is associated with reduced lean body mass, increased fat mass, reduced physical function, reduced cognitive function, increased depressive symptoms and increased risk of falls and bone fractures [26]. In our study, we therefore aim to assess whether there are differences in frailty between metastatic (mPCa) and localized prostate cancer under the effects of androgen blockade. Few studies to date have demonstrated differences in frailty syndrome between patients with mPCa and localized prostate cancer. A scoping review which analyzed the prevalence of frailty syndrome in mPCa found that 70% of patients with mPCa were frailer than localized patients [27]. We also propose to evaluate the role of other geriatric variables such as depressive symptoms and insomnia, given that studies have linked androgen depletion to depressive symptoms and/or insomnia in men without prostate cancer [28,29,30,31] and in patients with prostate cancer [32,33,34]. Since frailty has been related to age, polypharmacy, and overweight, we also analyzed the contribution of these variables.

## 2. Materials and Methods

### 2.1. Design and Study Population

A cross-sectional study was carried out between 2020 and 2022. The study included men diagnosed with metastatic and localized prostate cancer who had undergone prostatectomy, radiation therapy, and were receiving drugs acting as luteinizing hormone-releasing hormone (LHRH) analogues, such as androgen-deprivation therapy. After the measurements performed in this study, patients with metastatic diseases received additional androgen-deprivation therapies (enzalutamide, abiraterone, or apalutamide) according to the new clinical guidelines. Recruitment and evaluation were carried out by the Department of Urology at an Oncology Center (Department of Urology Oncology, IVO Foundation, Valencia, Spain, and the Oncology Service of the Doctor Peset University Hospital). The exclusion criteria were not being able to correctly understand the Spanish language or the content of the questions asked in the psychological and functional assessment. A total of 63 men agreed to participate and signed the informed consent form.

### 2.2. Frailty Syndrome Assessment

Frailty syndrome was assessed according to the frailty physical phenotype proposed by Fried et al.: (1) weight loss, defined as an unintentional loss of 4.5 kg or more in the last year; (2) self-reported exhaustion assessed by the question “How often in the last week did you feel that everything you did led to fatigue?”, with the criterion being met when participants answered “always” or “often”; (3) decreased physical activity, assessed with the International Physical Activity Questionnaire. The total amount of energy expended in minutes of activity over a week was calculated and the criterion was met when individuals’ physical activity was in the lowest quintile; (4) slowness in walking, calculated using the 4.6 m walking time, and the frailty criterion was considered when the participant was in the lowest quintile, after adjusting for height (7 s for participants with a height ≥1.73 m, and 6 s for those with a height <1.73 m); (5) muscle weakness, measured by establishing grip strength by quintiles, measured at baseline three times alternately for each hand, using a digital hand-held dynamometer. The last quintile recorded in the sample was considered a positive criterion. Based on Fried’s criteria, individuals were classified as pre-fragile if they met one or two criteria, or fragile if they met three or more. Participants who did not meet any criteria were classified as robust.

### 2.3. Geriatric Assessment

The mini-mental examination scale (MMSE) scale is a widely used scale to assess cognitive function and screen for dementia. It can be used by doctors, nurses, or researchers with minimal training, and takes about 10 min. The Mini Cognitive Exam (MEC) is the validated Spanish version of the MMSE and consists of 11 items that detect cognitive impairment by evaluating 5 cognitive areas: orientation (temporal and spatial), attention and calculation, word recall, language, and visuospatial skills. The maximum score is 30 points. A higher score indicates better cognitive function, and cut-off points of 23/24 have typically been used to show significant cognitive impairment [35].

The AIS is a self-report questionnaire used to screen for sleep disorders. Based on the International Classification of Diseases (ICD-10), the full scale is made up of eight items. The first five factors are related to nocturnal sleep and three factors are related to daytime dysfunction, with a score range of 0 to 24 (the cut-off point is six, with higher scores suggesting a more serious problem). It was validated by Soldatos et al. in 2000 [36] and validated in Spanish by Gómez-Benito et al. in 2011 [37]. 

The Abbreviated Geriatric Depression Scale (GDS) is a screening questionnaire developed by Yesavage et al. [38]. It consists of 15 items and was validated in Spanish by Martínez de la Iglesia et al. [39], with acceptable psychometric properties and a cut-off point of five or more. The Cumulative Geriatric Illness Score Scale (CIRS-G) was used to assess the comorbidity index [40].

## 3. Results

### 3.1. Sociodemographic and Clinical Data

This study included 63 patients diagnosed with metastatic and localized prostate cancer. The mean age of the participants was 73.6 ± 1.18 years (SEM) (age range 51–92 years), and all were men living in the community. As regards marital status, the patients were married (85.7%), divorced (4.8%), separated (6.3%) or widowed (3.2%). The mean time with androgen blockade was 72.8.3 ± 8.65 (SEM) (range 1–211 months). According to international PCa staging, at the time of diagnosis, the patients had stage I and II cancer (1.6% and 44.4% respectively), stage III cancer (14.3%), or stage IV cancer (38.1%) with bone or glandular metastases, and a mean Gleason score of 7.30 ± 0.14 (SEM) (range 5–10 points). The mean PSA ng/mL before starting androgen blockade was 16.7 ± 9.08 (SEM) (range 0.10–509). At the time of the study, more than half of the patients (61.9%) had undergone prostatectomy, while 38.1% had not. The mean BMI of the participants was 27.6 ± 0.42 (SEM) (range 19.6–34.5), and according to these data 23.8% of the patients had a “normal weight” (BMI = 18.5–24.9 kg/m^2^), while 54% were “overweight” (BMI = 25–29.9 kg/m^2^) and 22.2% were “obese” (BMI > 30 kg/m^2^). None of the patients were underweight (BMI <18.5 kg/m). A total of 58.7% of the patients had central or android obesity, with a waist circumference ≥102 cm. The age-adjusted Charlson index indicated a mean index of 2.87 ± 0.20 (SEM) (Range 0–7) as shown in Table 1.

### 3.2. Evaluation of Frailty Syndrome

**Figure 1:**
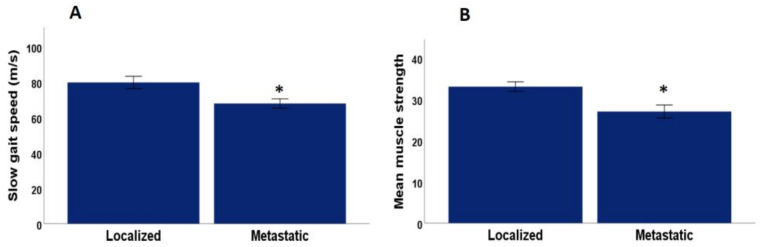
(**A**) Differences in gait speed between patients with mPCa and localized PCa. (**B**) Muscle strength differences between mPCa and localized PCa. * *p* < 0.05 significant difference between localized and mPCa.

The mean number of frailty criteria met in the sample was 1.46 ± 0.13 (SEM) (range 0 to 4). The frequency of each of the five criteria is specified in Table 2. Fifteen men were robust, i.e., they did not meet any frailty syndrome criteria (24.6%), 16 (25.4%) met one frailty criterion, 22 (34.9%) met two frailty criteria, eight (12.7%) met three frailty criteria, and two (3.2%) met four frailty criteria. The prevalence of two of the five frailty criteria (slow gait speed and low muscle strength), as shown in Figure 1, was statistically significant among the metastatic and localized patients. A total of 59.2% of the metastatic patients met the frailty criterion of a slow gait, while in the localized group the figure was 11.1% (*p* = 0.001, Chi-square test). A total of 14.8% of the metastatic patients presented reduced muscle strength, while 2.7% of the localized patients did (*p* = 0.04, Chi-square test). The other criteria turned out not to be significant, as shown in Table 2. 

### 3.3. Relationship between Frailty Syndrome and Socio-Demographic and Clinical Variables

Since the category of frail men was small (N = 10), we grouped pre-frail and frail men into one group, and called it the “pre-frail/frail” group, and we thus had robust and pre-frail/frail groups. There were no significant differences between the robust and pre-frail/frail groups in age (*p* = 0.89, Mann–Whitney U test), Charlson Index (*p* = 0.89, Mann–Whitney U test), Gleason Index (*p* = 0.86, Mann–Whitney U test), androgen blockade time (*p* = 0.73, Mann–Whitney U test), BMI (*p* = 0.35, Mann–Whitney U test), waist circumference (*p* = 0.09, Mann–Whitney U test), PSA values (ng/mL) (*p* = 0.81, Mann–Whitney U test), testosterone (ng/mL) (*p* = 0.53, Mann–Whitney U test), systolic blood pressure (*p* = 0.73, Mann–Whitney U test), diastolic pressure (*p* = 0.36, Mann–Whitney U test), or prostatectomy (*p* = 0.13, Chi square test). Since age could influence severity of frailty syndrome, we also categorized the ages of patients as 65 years old or 75 years old in order to analyze whether there were significant relationships with the number of Fried criteria. There were no significant differences with categorized age ≥ 65 years (*p* = 0.66, Mann–Whitney U test) and categorized age ≥75 years (*p* = 0.67, Mann–Whitney U test). Likewise, there was no significant relationship between frailty categories e.g., robust, pre-frail or frail patients with categorized age >65 years (*p* = 0.49, Chi square test) and categorized age >75 years (*p* = 0.73, Chi square test). 

**Figure 2:**
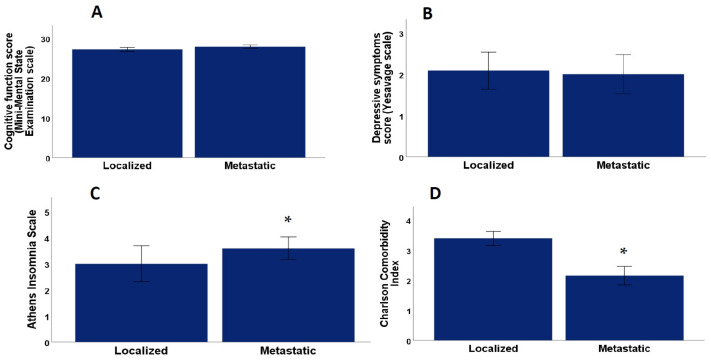
(**A**) Differences in Mini-Mental State Examination Scale score between mPCa and localized PCa. (**B**) Yesavage Scale score between mPCa and localized PCa (**C**) Athens Scale score between mPCa and localized PCa and (**D**) differences in Charlson Index between mPCa and localized PCa. * *p* < 0.05 significant difference between localized and mPCa.

**Figure 3:**
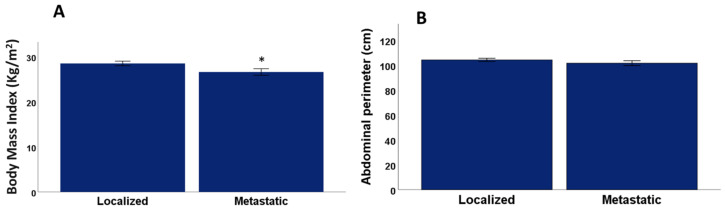
Differences in Body Mass Index (**A**) and abdominal perimeter (**B**) between mPCa and localized PCa. * *p* < 0.05 significant difference between localized and mPCa.

There were no significant differences between metastatic and localized patients for age (*p* = 0.37, Mann–Whitney U test), Gleason Index (*p* = 0.21, Mann–Whitney U test). Significant differences were found between metastatic and PCa localized patients with a mean Charlson Index value for metastatic patients of 2.62 ± 0.29 (SEM), and a mean value for PCa localized patients of 3.39 ± 0.23 (SEM) (*p* = 0.02, Mann–Whitney U test) as shown in Figure 2. Likewise, the androgen blockade time had a mean value for the metastatic group of 54.0 ± 13.5 (SEM) and a mean value for the PCa localized group of 105.1 ± 15.4 (SEM) (*p* = 0.004, Mann–Whitney U test), BMI had a mean value for metastatic patients of 26.2 ± 0.62 (SEM) and a mean value for PCa localized patients of 28.5 ± 0.51 (SEM) (*p* = 0.01 Mann–Whitney U test) as shown in Figure 3. There were also significant differences in the PSA ng/mL values, with a mean value for the metastatic group of 38.1 ± 24.2 (SEM) and a mean value for the localized group of 4.26 ± 1.04 (SEM) (*p* = 0.04, Mann–Whitney U test), systolic blood pressure with a mean value for metastatic group 143.03 ± 2.7 (SEM) and mean value for the localized group 134.7 ± 2.6 (*p* =0.03, Mann–Whitney U test)). No significant differences were found between the metastatic and PCa localized patients for diastolic blood pressure (*p* = 0.08, Mann–Whitney U test), abdominal circumference (*p* = 0.16, Mann–Whitney U test), testosterone values ng/mL (*p* = 0.89, Mann–Whitney U test) and prostatectomy (*p* = 0.47, Chi-square test). 

### 3.4. Relationship between Frailty Syndrome and Geriatric Assessment

There were no significant differences between the robust and pre-frail/frail groups on the Athens Scale (*p* = 0.06, Mann–Whitney U test). The numbers of frailty criteria differed significantly among people with poor sleep quality with a mean value of 2.57 ± 0.36 (SEM) compared to those who did not have insomnia problems, of 1.33 ± 0.14 (*p* = 0.008, Mann–Whitney U test). No significant differences were found between the robust and pre-frail/frail groups for MMSE and its five domains: temporal orientation (*p* = 0.22, Mann–Whitney U test), spatial orientation (*p* = 0.18, Mann–Whitney U test), fixation (*p* = 0.56, U test), attention calculation (*p* = 0.55, Mann–Whitney U test), delayed recall (*p* = 0.28, Mann–Whitney U test), language (*p* = 0.07, Mann–Whitney U test), and the total MMSE score (*p* = 0.18, Mann–Whitney U test). Next, we analyzed whether age influences having a better or worse score on the Mini-mental test score, but no significant differences were found between categorized age >65 years with the total score of the Mini Mental (*p* = 0.48, Mann–Whitney U test). In the same way, there was no significant relationship between categorized age >75 and the Mini-Mental test score (*p* = 0.21, Mann–Whitney U test). There were no significant differences with age and the self-rated Health Perception Scale (*p* = 0.62, Mann–Whitney U test) and Yesavage Scale (*p* = 0.70, Mann–Whitney U test).

Significant differences were found between the metastatic and localized patients on the Athens Scale, with a mean value for metastatic patients of 3.59 ± 0.43 (SEM) and a mean value for localized patients of 3.00 ± 0.69 (*p* = 0.02, Mann–Whitney U test) as shown in Figure 2. There were no significant differences between the metastatic and localized patients on the MMSE for temporal orientation (*p* = 0.66, Mann–Whitney U test), spatial orientation (*p* = 0.96, Mann–Whitney U test), fixation (*p* = 0.23, Mann–Whitney U test), attention and calculation (*p* = 0.89, Mann–Whitney U test), delayed recall (*p* = 0.86, Mann–Whitney U test), language (*p* = 0.16, Mann–Whitney U test), or the total MMSE score (*p* = 0.48, Mann–Whitney U test) on the Health Status Perception Scale (*p* = 0.40, Mann–Whitney U test), or on the Yesavage Scale (*p* = 0.84, Mann–Whitney U test).

## 4. Discussion

Few studies have compared the level of frailty between patients with metastatic vs. localized PCa [41,42,43,44,45,46,47]. A recent review analyzed frailty syndrome in patients with mPCa [27], showing a prevalence between 30–70% depending on the geriatric assessment tool used. Frailty was also identified as associated with other CGA assessments and quality of life assessment results, with mCaP patients being frailer and showing poorer HRQoL compared to those without metastases. In general, the CGA scores for patients with mPCa were lower than those for patients without metastases. In our study involving a population of community-dwelling men diagnosed with mPCa and localized PCa who had undergone treatment with prostatectomy and androgen deprivation, we identified approximately 18.5% of the patients with mPCa as frail (fulfilling three or more criteria), 63% as prefrail (fulfilling at least one or two criteria), and 18.5% as robust (no frailty criteria), compared to the localized PCa group, which was 13.8% frail, 58.3% pre-frail, and robust 27.7%. Therefore, the number of frailty patients with mPCa and localized PCa was similar, however, the number of prefrail patients with mPCa was higher compared to the localized PCa group. Due to the limited sized of the study it is not possible to demonstrate that mPCA group present higher prevalence of prefrailty or frailty. However, the results, reported for the first time, that two frailty criteria were more altered in mPCa patients which can have important consequences for personalized tailored healthcare programs in this group.

Due to the limited sized of the study it is not possible to demonstrated that mPCA group present higher prevalence of prefrailty or frailty; however, the results clearly showed that two frailty criteria were more altered in mPCa patients, which may have important consequences for personalized tailored healthcare programs in this group. The age-related decline in physical function and increased prevalence of frailty parallels the androgen decline seen in older men, which has a detrimental effect on several aspects of male health [48]. In addition, symptoms associated with androgen deprivation therapy for prostate cancer (e.g., sarcopenia or loss of lean mass, muscle weakness, fatigue, and decreased activity levels) are also components of the frailty syndrome [49]. 

Multimorbidity has been shown to be related to frailty, although it is not synonymous with it [50]. Comorbidities are common in cancer patients and their prevalence increases with age. Comorbid conditions act as confounding factors that complicate cancer diagnosis and treatment, mediate the effects of cancer and cancer treatment, and pose competing risks of morbidity and mortality [51]. In our study, metastatic patients had fewer comorbidities than patients with localized disease, so the higher frailty in two of the five Fried criteria (decreased muscle strength and decreased gait speed) could not be attributed to an increased burden of comorbidities. There is evidence from studies that have analyzed in patients with PCa that both age and a greater number of comorbidities have been associated with a higher prevalence of frailty [52]. Metastatic cancer is a systemic disease that affects multiple organs and systems of the body. Although the pathophysiology of frailty is not fully understood, some studies have proposed chronic inflammation as an underlying biological mechanism responsible for decreased physical function in older people, and it has been associated with increased morbidity and mortality in older people [53]. In general, the relationship between comorbidities and metastatic cancer is complex, and may be influenced by a variety of factors. More research is needed to fully understand the relationship between the two factors. 

The results of our study have shown a trend of physical frailty in two of the five Fried criteria, e.g., decreased muscle strength and slow walking speed, for patients with mpCa compared with patients with localized PCa. Age-related loss of muscle mass and strength, known as sarcopenia, may be further complicated by the development of cancer and cancer-related treatments [54]. Worldwide, sarcopenia is now recognized as a clinical disease characterized by variable combinations of slow walking speed, weak muscle strength, and low lean appendicular mass [55]. Low skeletal muscle mass is highly prevalent in older adults with cancer and is strongly associated with increased risk of adverse events, such as chemotherapy-related toxicities, ADT use, surgical complications, and decreased overall survival [56,57]. In our study, we found that 14.8% of patients with mPCa had reduced muscle strength compared to 2.7% in the group of localized PCa, based on the cut-off value for this frailty criterion. Evidence from previous studies has shown that muscle strength assessed by manual dynamometry was 29% lower in prostate cancer patients receiving ADT than in the age-matched disease-free control group [58]. A cross-sectional study evaluating the effect of ADT on lean body mass (LBM), muscle strength, bone mineral density, sexual function, and quality of life (QOL) in men with PCa found that androgen-deprived men had increased fat mass and reduced upper and lower body strength compared to control groups. This demonstrated that the reduced strength in the ADT group was due to hypogonadism, and not an effect of the disease (PCa) itself [59]. The slowing of the gait in patients with metastatic prostate cancer may be due to several factors. Bone metastasis, which is common in advanced prostate cancer, can cause muscle pain and weakness, which can affect the patient’s ability to walk [60]. The results of this study have shown that 59.2% of the patients with mPCa had a slower walking speed compared to 11.1% in the group with localized PCa based on the cut-off value for this frailty criterion. In recent years, there has been increased interest in measuring patients’ walking speed at habitual pace (gait speed) over short distances, to detect frailty [61]. Evidence from previous studies has shown that a slow walking speed of more than 4 m predicts mortality and the occurrence of medical problems (hospitalization, disability, falls, cognitive decline) in older people living in the community [62,63]. In addition, significant decreases in gait speed have been shown before cancer diagnosis, specifically in people with prostate cancer since many of these people probably had asymptomatic and localized disease. Gait speed turns out to be a potential marker of frailty and as a critical tool in risk prediction in older adults with cancer due to its association with higher mortality and disability [56]. Further studies are needed to examine the interaction between muscle loss and muscle weakness and adverse health-related outcomes.

In this study, significant differences were found between patients with mCaP and localized CaP and BMI. A total of 32.1% of patients with mPCa were normal weight, 51.8% overweight, and 14.8% obese. On the other hand, patients with localized PCa 16.6% had a normal weight, 55.5% were overweight and 27.7% were obese. However, we found no significant relationship between frailty and BMI. Although patients will almost certainly lose muscle mass, ADT leads to overall weight gain rather than weight loss, and the commonly used Fried Biological Syndrome model probably underestimates frailty in older men receiving ADT due to their weight loss criteria. Lean weight loss, or sarcopenia, has been suggested as being the relevant factor in the development of frailty [64]. In addition, obesity is also known to be associated with a higher probability of pre-frailty and frailty [65]. We believe that increased BMI due to changes in body composition is an integral part of the frailty syndrome in patients receiving ADT. Some studies have argued that “weight loss” is not a necessary component of frailty, as obesity has been shown to be associated with frailty syndrome, including its negative outcomes [66]. A study evaluating obese frailty in patients with PCa raised the issue of using the modified Fried criteria, since inducing obesity in men receiving ADT would put them at risk of “obese frailty.” They therefore created a modified frailty criterion, substituting “obesity” for the original criterion of “involuntary weight loss”, and retaining the same number of positive criteria needed as the original definition [67]. It is vitally important that future frailty studies in men with PCa receiving ADT consider both the loss of lean muscle mass and the increase in adiposity when assessing physical frailty using the criteria proposed by Fried.

It should also be emphasized that a significant association was found between metastatic and localized patients with insomnia symptoms in our study. The patients with mPCa had higher scores than the PCa localized group and presented poorer sleep quality. Prostate cancer treatment is often associated with nocturia, defined as the need to urinate more than twice a night, as the most common cause of sleep disturbances [68]. In addition, many prostate cancer patients receive hormone therapy that can induce hot flashes and night sweats associated with sleeping difficulties [69]. Hot flashes are commonly present in patients treated with ADT, reported in about 80% of patients, and 27% of patients consider it the most striking adverse effect [70]. Patients with insomnia are prone to emotional disturbances, chronic fatigue, poor professional performance, and dependence on sedatives [71]. There is evidence to suggest a strong correlation between poor sleep quality and profound negative outcomes, including reduced physical and psychological functioning, and poorer quality of life [69]. Prospective studies are required in order to evaluate the impact of sleep disturbances in patients with mPCa and its relationship with frailty syndrome.

## 5. Conclusions

Individuals with mPCa had higher physical frailty odds compared to individuals with localized prostate cancer under androgen-deprivation theory, and, in particular, this group displayed a reduced muscular strength and reduced walking speed. Therefore, there is a need for increased guidance by healthcare professionals to encourage people with prostate cancer and mPCa to maintain or increase levels of physical activity after diagnosis to maintain or improve an adequate muscular strength and speed walking under androgen-deprivation therapy. Given the individualized needs of these patient groups, referral to a specialist for the prescription of personalized and supervised physical activity programs should be considered. In fact, there is evidence from studies that have evaluated the intervention of a resistance, aerobic, and flexibility multimodal exercise program both in patients with mPCa and in patients with localized PCa, and have shown that there is an improvement in physical function, muscle strength, and also an improvement in mental health [72,73,74]. The higher levels of frailty in this group cannot be explained by older age, higher comorbidity index, longer time under androgen-deprivation therapy, or more prevalence of overweight/obesity. This study provides novel data in order to provide personalized tailored healthcare programs for these patients based on these deficits. The presence and the role of systemic factors associated with metastatic disease that lead to increase frailty need to be investigated in future studies. 

## Figures and Tables

**Table 1:** Clinical characteristics of patients enrolled in the study.

Variables	Frequency % (Categorical Variables) or Mean ± Standard Error of the Mean (Range Min–Max) (Discrete Variables)	mPCa Patients (N = 27)	PCa Localized (N = 36)	*p* Value
Previous prostatectomy				0.36
Yes	38 (61.9%)	Yes (51.9%)	Yes (66.7%)	
No	25 (38.1%)	No (48.1%)	No (33.3%)	
BMI (kg/m^2^)				0.22
Underweight (<18.5)	0			
Normal (18.5–24.9)	15 (23.8%)	9 (32.1%)	6 (16.6%)	
Overweight (25–29.9)	34 (54%)	14 (51.8%)	20 (55.5%)	
Obese (>30)	14 (22.2%)	4 (14.8%)	10 (27.6%)	
Abdominal perimeter				0.65
<102 cm	26 (41.3%)	12 (44.4%)	14 (38.8%)	
≥102 cm	37 (58.7%)	15 (55.5%)	22 (61.1%)	
Age	73.6 ± 1.18 (51–92)	75 ± 1.6	72.9 ± 1.6	0.42
Gleason Index	7.30 ± 0.14 (5–10)	7.5 ± 0.25	7.1 ± 0.17	0.21
Charlson Comorbidity Index	2.87 ± 0.20 (0–7)	2.6 ± 0.29	3.3 ± 0.23	0.02
Body mass index	27.6 ± 0.42 (19.6–34.5)	26.6 ± 0.73	28.5 ± 0.51	0.01
Abdominal perimeter	102.8 ± 1.08 (84–125)	101.8 ± 2.0	104.3 ± 1.33	0.28
Criteria of frailty syndrome				0.57
Robust (0 criteria)	15 (23.8%)	5 (18.5%)	10 (27.7%)	
Pre-frail (1–2 criteria)	38 (60.3%)	17 (63%)	22 (58.3%)	
Frail (>3 criteria)	10 (15.9%)	5 (18.5%)	5 (13.8)	
Number of Fried criteria	1.46 ± 0.13 (0–4)	1.55 ± 0.20 (0–4)	1.38 ± 0.18 (0–4)	0.78
Prevalence of prefrailty plus frailty	76.20%	81.50%	72.30%	0.76
Athens Scale	3.26 ± 0.43 (0–17)	3.59 ± 0.43	3.0 ± 0.69	0.02
MMSE Scale	27.5 ± 0.34 (16–30)	27.81 ± 0.43	27.2 ± 0.50	0.42
Yesavage Scale	2.05 ± 0.32 (0–14)	2.0 ± 0.47	2.09 ± 0.44	0.67
Health Perception Scale	7.51 ± 0.20 (2–10)	7.70 ± 0.32	7.37 ± 0.26	0.43

**Table 2:** Criteria of frailty syndrome in metastatic and localized patients.

	Prevalence %	*p* Value for the Comparison between Metastatic and Localized PCa
1. Frailty criterion: involuntary weight loss	Yes 22 (34.9%)	0.76
	No 41 (65.1%)	
2. Frailty criterion: weakness	Yes 17 (27%)	0.11
	No 46 (73%)	
3. Frailty criterion: low physical activity	Yes 28 (44.4%)	0.11
	No 35 (55.6%)	
4. Frailty criterion: slow gait speed	Yes 19 (31.1%)	0.001
	No 42 (68.9%)	
5. Frailty criterion: low muscle strength	Yes 5 (7.9%)	0.04
	No 58 (92.1%)	

## Data Availability

The raw data supporting the results of this paper will be made accessible upon request by the corresponding author based on scientific purposes.
